# Long-term cancer-related fatigue outcomes in patients with locally advanced prostate cancer after intensity-modulated radiotherapy combined with hormonal therapy

**DOI:** 10.1097/MD.0000000000003948

**Published:** 2016-06-24

**Authors:** Hua-Chun Luo, Yong Lei, Hui-Hua Cheng, Zhi-Chao Fu, Shao-Guang Liao, Jing Feng, Qin Yin, Qun-Hua Chen, Gui-Shan Lin, Jin-Feng Zhu, Jian-Feng Xu, Dian Wang

**Affiliations:** a Department of Radiation Oncology, FuZhou General Hospital of NanJing Command PLA, FuZhou; b Department of Medical, FuZhou General Hospital of NanJing Command PLA, FuZhou; c Department of Mendicine, LongYan Hospital of Tradiational Chinese Medicine, LongYang; d Department of Radiation Oncology, FuJian Province Hospital, FuZhou; e Department of Rrology, JinJiang Hospital, QuanZhou, China; f Department of Radiation Oncology, Rush University Medical Center, Chicago, IL.

**Keywords:** cancer-related fatigue, prostate cancer, quality of life, radiotherapy

## Abstract

The aim of our study was to investigate the relationship between cancer-related fatigue and clinical parameters, and the effect factors of fatigue for the prostate cancer patients. Long-term follow-up is performed using the Fatigue Symptom Inventory before treatment (A), at the end of intensity-modulated radiotherapy (B), and 3 months (C), 12 months (D), 24 months (E), 36 months (F), and 48 months (G) after the end of intensity-modulated radiotherapy. Three dimensions of fatigue are assessed during follow-up: severity, perceived interference with quality of life, and duration in the past week. In all, 97 patients with locally advanced prostate cancer were enrolled in the study. Median follow-up time was 43.9 months. The fatigue index was significantly higher in the prostate-specific antigen >20 ng/mL, Gleason score >8, the Eastern Cooperative Oncology Group scores, and the higher education. The most severe fatigue occurred at time points B and C. The score for duration of fatigue fluctuated across the time points, with significantly increased scores at time points D, E, and F.

In conclusion, we show that cancer-related fatigue is the important symptom which affects the quality of life for the prostate cancer patients. For patients with locally advanced prostate cancer with a high Eastern Cooperative Oncology Group score, a Gleason score of >8 points, prostate-specific antigen levels of >20 ng/mL, and high education, attention should be paid to the interference of fatigue with quality of life, especially general level of activity, ability to concentrate, and mood, after radiotherapy combined with hormonal therapy.

## 1 Introduction

Prostate cancer is the most frequent malignant cancer in American men, and also has an increasing incidence in developing countries, including China.^[1,2]^ The survival rate of patients with locally advanced prostate cancer has been greatly increased with surgery, radiotherapy, and hormonal therapy as the primary therapies.^[2–4]^ Quality of life (QOL) has become an important factor for treatment decision-making.^[5]^ Our previous follow-up of patients with locally advanced prostate cancer for up to 8 years after radiotherapy combined with hormonal therapy^[6]^ found that fatigue was an important clinical symptom affecting QOL. The questionnaire can be used as a primary investigation method to make intuitive and effective analysis of survival in patients with prostate cancer.^[7]^ Fatigue has not been investigated in previous studies on QOL in patients with prostate cancer after radiotherapy.^[6,8,9]^ In the present study, patients receiving radiotherapy combined with hormonal therapy were followed up by questionnaire, to evaluate the effect of this treatment on fatigue, and to investigate the relationship between cancer-related fatigue (CRF) and clinical parameters.

## 2 Methods

### 2.1 Inclusion criteria

Patients with prostate cancer, confirmed by histopathology, who were admitted to Fuzhou General Hospital of Nanjing Military Command from February 2008 to December 2012, were selected. Among them, those who had an Eastern Cooperative Oncology Group (ECOG) score ≤2 points, no distant organ metastasis confirmed by imaging, and no previous history of cancer, and met any of the following criteria, were included: (1) a Gleason score of 8 to 10 points; (2) serum prostate-specific antigen (PSA) of ≥20 ng/mL; (3) prostate cancer staged as T3 or T4 by magnetic resonance imaging (MRI) (tumor penetration of prostatic capsule or tumor invasion of other adjacent structures outside the seminal vesicle, such as the bladder neck, external sphincter, rectum, levator ani muscle, and pelvic wall), with or without regional lymph node metastasis.

Approval for the study was given by the FuZhou General Hospital Research Ethics Committee. Eligible men were identified from the clinic 7 days before their appointment.

### 2.2 Radiotherapy

Supine position was used. Computed tomography (CT)-enhanced scan images (2.5 mm thick) were ranged from the edge of L2 to 10 cm below the edge of sciatic. The CT-scan images and pelvic MR images fused together. Clinical target volume included entire prostate, seminal vesicle, and pelvic lymph node drainage area. The pelvic lymph node drainage area was defined according to the recommendation of Radiation Therapy Oncology Group (RTOG). Gross tumor volume (GTV)_nd_ was defined as pelvic lymph node (minor axis≥1.0 cm). The following normal organs were also delineated: rectum, bladder, femoral head, small intestine, and colon. A total dose of PTV and GTV_nd_ were 72.6 Gy/2.2 Gy/6+w. A total dose of prophylactic irradiation of the pelvic lymph node drainage area was 56.1 Gy/2.2 Gy/6+w.

### 2.3 Hormonal therapy

Hormonal therapy was performed from the first day of intensity-modulated radiotherapy (IMRT) with 50 mg of oral Casodex once daily and 3.6 mg of Zoladex via subcutaneous injection every 28 days for 30 months.

### 2.4 Symptoms of fatigue

The Fatigue Symptom Inventory (FSI) was used.^[10]^ The questionnaire included 13 items on 3 dimensions: severity, perceived interference with QOL, and duration in the past week. Severity was measured by assessing most, least, and average fatigue in the past week, and also current fatigue. Perceived interference with QOL was measured by assessing interference with general level of activity, ability to bathe and dress, normal work activity, ability to concentrate, relations with others, enjoyment of life, and mood. Duration was measured as the number of days in the past week that respondents felt fatigued, and also the extent of each day on average they felt fatigued. A higher score indicates more severe fatigue. The questionnaire was administered before treatment (A), the day when IMRT ended (B), and 3 months (C), 12 months (D), 24 months (E), 36 months (F), and 48 months (G) after the end of IMRT.

### 2.5 Questionnaire administration and management of data loss

At time points A, B, and C, the questionnaire was completed by the doctor and patient together. At the other time points, the questionnaire was mailed to the patient. If a letter in reply was not received within 4 weeks, the questionnaire was completed by telephone. A questionnaire was excluded if none of the items in any dimension was scored. Patients who had organ metastasis or biochemical recurrence, were lost to follow-up, or died during follow-up were removed. If all items of the questionnaire were answered, scores for each dimension were calculated from the actual scores. If only part of the items were answered, scores for each dimension were calculated by averaging the nonmissing scores within the dimension.

### 2.6 Statistical methods

All data were analyzed using SPSS 13.0. Clinical data were presented as mean ± standard deviation (SD). Scores for each dimension in the FSI questionnaire were presented as mean values. Normality tests were performed using the chi-square test. Differences between groups were determined by the descriptive *t* test. Changes in each dimension were assessed compared with time point A. The rate of change in a single sample was compared using the Wilcoxon rank-sum test. The scores at each time point were compared with those at time point A using the Mann–Whitney test. Two-tailed *P* < 0.05 was considered statistically significant. Line charts were produced using Graphpad Prism 5.

## 3 Results

### 3.1 General information and questionnaire results

**Figure 1:**
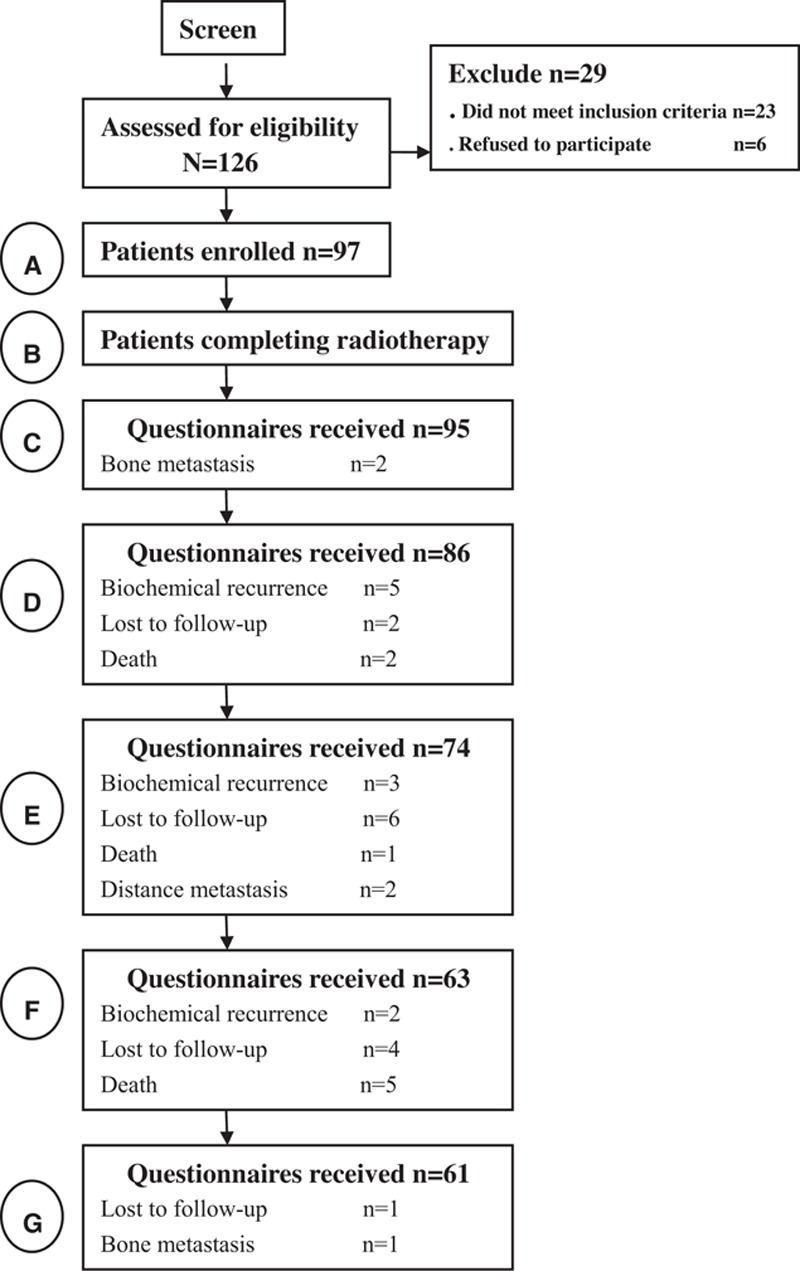
Diagram showing the study cohort according to received treatment. A = time A (base evaluate), B = time B (after radiation), C = time C (3 months), D = time D (12 months), E = time E (24 months), F = time F (42 months), G = time G (42 months).

A total of 126 patients with prostate cancer met the inclusion criteria were admitted to the hospital. Of them, 97 (76.9%) agreed to participate in the long-term questionnaire follow-up. General information and IMRT-related data of patients enrolled are shown in Table 1. The median follow-up time was 43.9 months (range 14.5–72.6). All patients completed the treatment. The number of valid questionnaires at each time point are listed in Table 2, and are as follows: C (n = 95): 2 patients with bone metastasis; D (n = 86): 5 with biochemical recurrence, 2 lost to follow-up, and 2 deaths; E (n = 74): 3 with biochemical recurrence, 6 lost to follow-up, 1 death, and 2 with distal metastasis; F (n = 63): 2 with biochemical recurrence, 4 lost to follow-up, and 5 deaths; G (n = 61): 1 lost to follow-up, and 1 with bone metastasis (Fig. 1).

**Table 1:**
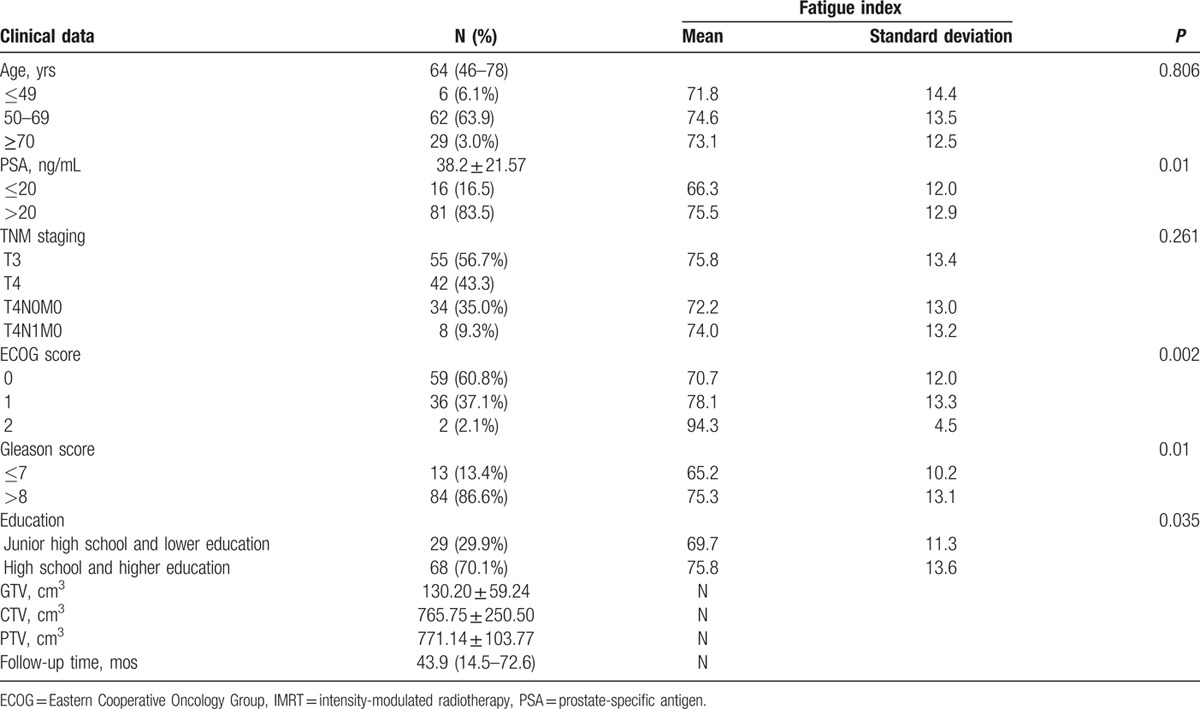
Clinical data and IMRT-related data of patients enrolled.

**Table 2:**
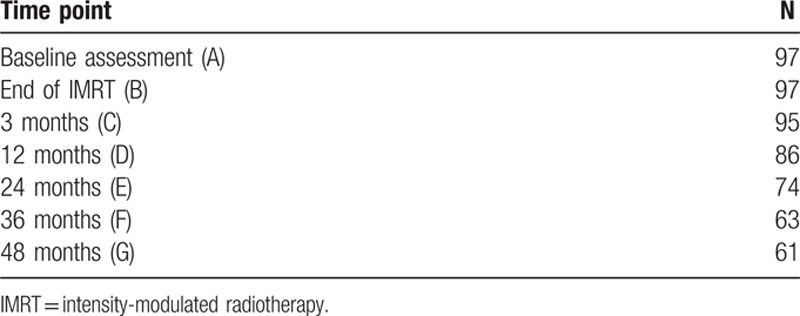
Number of valid questionnaires.

### 3.2 Relationship between cancer-related fatigue and clinical parameters

No significant difference was noted in baseline assessment among the 3 age groups (≤49, 50–69, and ≥70; *P* > 0.05). No significant difference was noted in fatigue index between stages T3 and T4 (*P* > 0.05). The fatigue index was higher with PSA >20 ng/mL than PSA ≤20 ng/mL, indicating that fatigue is associated with PSA levels. The ECOG score was significantly positively correlated with the fatigue index (*P* < 0.05). The higher the Gleason score and education level, the higher the fatigue index. The relationship between fatigue index and clinical parameters is shown in Table 1.

### 3.3 FSI scores at each time point

**Figure 2:**
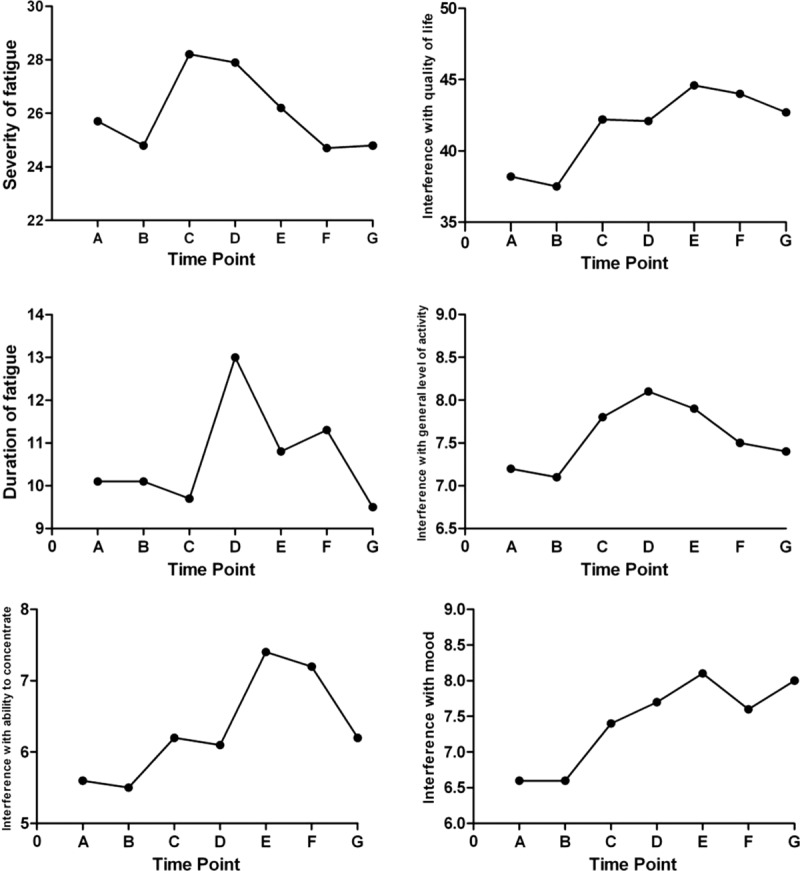
The scores for each dimension.

The severity of fatigue did not change significantly among follow-up time points, but the most severe fatigue mostly occurred 3 months and 1 year after the end of IMRT. Interference with QOL increased at each time point from 3 months after the end of IMRT to the end of follow-up and demonstrated significant differences from baseline score. In this dimension, the most severe interference with general level of activity occurred from 1 year after the end of IMRT to the end of hormonal therapy; ability to concentrate and mood became worse from the end of IMRT to the end of hormonal therapy; the most severe interference with normal work activity, relations with others, and enjoyment of life was observed at the end of hormonal therapy; and there was no significant fluctuation in ability to bathe and dress throughout the follow-up period. The most significant fluctuation in duration of fatigue was observed from 12 to 36 months; and the number of days of fatigue did not differ significantly among each time point. Results are shown in Table 3. The scores for each dimension, and also interference with general level of activity, ability to concentrate, and mood, are plotted in Fig. 2.

**Table 3:**
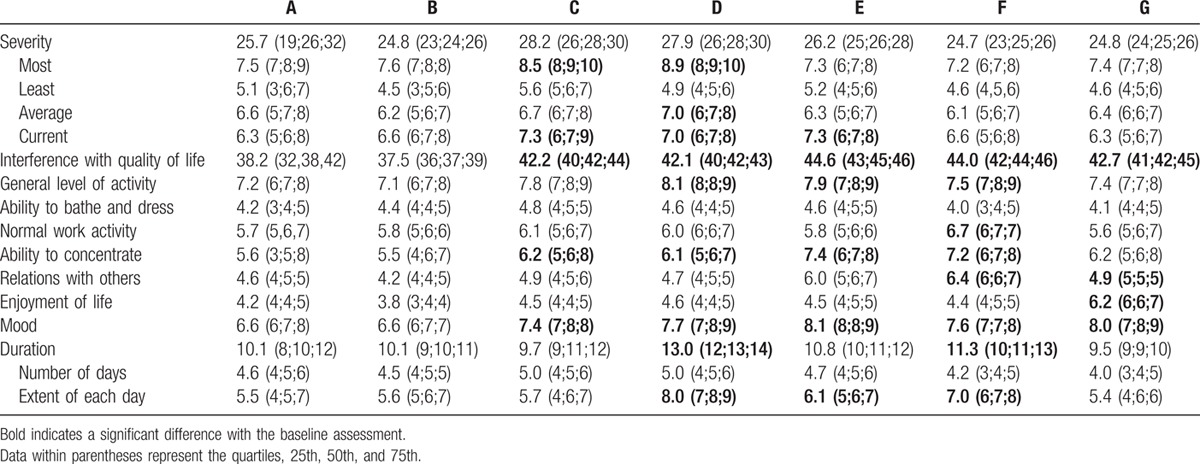
Fatigue Symptom Inventory scores at each follow-up time point.

## 4 Discussion

According to National Comprehensive Cancer Network (NCCN) 2014 Practice Guidelines for CRF, it is a distressing, persistent, subjective sense of physical, emotional, and/or cognitive tiredness or exhaustion related to cancer or cancer treatment that is not proportional to recent activity that interferes with usual functioning. It must be managed and assessed at the beginning of treatment.^[11]^ CRF occurs in cancer patients at an incidence of 50% to 90%.^[12]^ FSI has been verified for reliability and validity.^[13]^

Because of possible overdiagnosis,^[14]^ PSA should not be used as a routine diagnostic screening tool for patients aged <50 years and >75 years, although it can provide a survival benefit for older men.^[15–16]^ The median age of patients enrolled in this study was 64 years. Prostate cancer is mostly diagnosed in men aged >50 years, and locally advanced prostate cancer mostly presents at an older age. Age is a factor in development of prostate cancer, but it is not related to fatigue; and the higher the ECOG score, the more severe the fatigue, which is consistent with the conclusion of study by Blackhall et al.^[17]^ This indicates that fatigue is not related to body function degeneration, but merely is a subjective feeling. The ECOG score verifies the reliability of FSI indirectly. Physical condition is closely related to the fatigue index, and is also the main factor affecting QOL.^[18]^ Timely assessment and intervention of fatigue can improve physical condition, thus improving QOL.^[19]^ The prostate tumor burden increased with the progression of clinical stage, but no significant differences in the fatigue index was observed. However, there were significant differences in the stratified PSA levels and Gleason score. This is consistent with the findings about fatigue in breast cancer,^[20]^ but contrary to our previous findings about fatigue in nasopharynx cancer.^[21]^ The pathogenesis of fatigue in hormonal therapy-related tumors may be associated with hormone disorders. Solid tumors may be associated with physiological reactions caused by changes in tumor burden. It needs to be further verified whether CRF is related to disorders of inflammatory regulators and excessive release of inflammatory cytokines acting on the endocrine system,^[22]^ and whether severity of fatigue is related to androgen deprivation. Patients with higher education have more knowledge of prostate cancer, but do not deeply understand the pathogenesis and prognosis of the disease, which to some extent increases the possibility of fatigue. By contrast, patients with lower education have better treatment compliance, which, to some extent, reduces the psychological burden that may induce fatigue.

In general, 60% to 100% of cancer patients have varying degrees of fatigue, a higher incidence in those receiving active anticancer treatment.^[23]^ At present, studies on CRF focus on breast cancer^[24]^ and lung cancer, and the relevant large clinical research or meta-analysis focuses on how it can be treated.^[25–26]^ Few studies on prostate CRF have been reported, and interference of fatigue with QOL have not been reported. A questionnaire was administered to patients with locally advanced prostate cancer at selected time points to investigate the interference of fatigue with QOL after radiotherapy combined with hormonal therapy. The severity of fatigue did not change significantly among each time point, but the most severe fatigue was observed 3 months and 1 year after the end of IMRT.

Our previous study found that hormonal function was a main factor affecting QOL in the first year after concurrent chemoradiotherapy.^[6]^ Androgen deprivation may increase the severity of fatigue. It needs to be further verified whether the development of these fatigue-related symptoms is related to dysregulation of cortisol secretion after androgen deprivation.^[27]^ The patients enrolled experienced serious interference with QOL, especially general level of activity, ability to concentrate, and mood, from 1 year after treatment, until the end of follow-up. They experienced fluctuations in general level of activity, ability to concentrate, and mood due to the treatment of disease itself, and also hormonal dysfunction caused by castration level of testosterone after hormonal therapy. Intermittent hormonal therapy seems to provide a better QOL, including mood, compared with continuous hormonal therapy.^[28]^ More clinical data are required to investigate whether IMRT combined with intermittent hormonal therapy can improve fatigue, including changes in the FSI items.

### 4.1 Limitations

The FSI mainly assesses interference of fatigue with QOL in the past week. Precise assessment of fatigue cannot be made during the long-term survival of patients with prostate cancer due to the poor timeliness and limited follow-up time points. The FSI items describe the specific impact of fatigue only, but do not allow stratification of fatigue. In addition, a total of 127 questionnaires were not fully completed during the follow-up of 48 months, which caused a deviation in the assessment of fatigue.

## 5 Conclusions

For patients with locally advanced prostate cancer with a high ECOG score, a Gleason score of >8 points, PSA levels of >20 ng/mL, and high education, attention should be paid to the interference of fatigue with QOL, especially general level of activity, ability to concentrate, and mood, after radiotherapy combined with hormonal therapy.
